# Feasibility of reduced setup uncertainty in intensity-modulated proton therapy for mediastinal lymphoma

**DOI:** 10.1016/j.phro.2026.100953

**Published:** 2026-03-22

**Authors:** Pietro Pisciotta, Adriaan Hengeveld, Erik W Korevaar, Sabine Visser, Dirk Wagenaar, Petra Klinker, John H. Maduro, Johannes A Langendijk, Anne GH Niezink, Stefan Both

**Affiliations:** University of Groningen, University Medical Center Groningen, Department of Radiation Oncology, Groningen, Netherlands (the)

**Keywords:** Lymphoma, Proton therapy, Robustness, 3DREM, 4DREM

## Abstract

**Background and purpose:**

Mediastinal lymphoma patients are typically young and are at risk of long-term radiation-induced adverse events, making organ of interest (OOI) sparing a key objective of radiotherapy. This study investigated whether reducing setup uncertainty during robust intensity-modulated proton therapy (IMPT) planning is feasible without compromising target coverage and delivered dose robustness.

**Materials and methods:**

Ten consecutive mediastinal lymphoma patients treated with IMPT were retrospectively analyzed. Treatment plans were generated on the four-dimensional average computed tomography (4DCT) using a 5 mm setup uncertainty (clinical standard). Additional plans with 4 and 3 mm setup uncertainties were created using a dose-mimicking optimization approach while maintaining identical clinical priorities. Robustness was evaluated using a three-dimensional robustness evaluation method (3DREM) and a longitudinal four-dimensional robustness evaluation method (4DREM), incorporating setup and range variations, machine uncertainties, respiratory motion, and weekly anatomical changes.

**Results:**

All plans achieved adequate target coverage in the 3D-nominal and 4DREM voxel-wise mean dose distributions (V_95 %_ > 99.6 %). The voxel-wise minimum dose distributions from 4DREM confirmed robust target coverage (V_95 %_ > 98 %, D_98 %_ > 95 % of prescription dose) for all setup uncertainties, with one clinically accepted exception. Reducing setup uncertainty resulted in statistically significant reductions in organs of interest doses (p < 0.01), corresponding to median reductions in predicted lifetime acute coronary event risk of 0.17 % and 0.32 % for 4 mm and 3 mm setup uncertainties, respectively (p < 0.01).

**Conclusion:**

Reducing setup uncertainty during robust IMPT planning for mediastinal lymphoma patients was feasible and improved OOI sparing without compromising delivered dose robustness.

## Highlights

• Reducing setup uncertainty to 3 mm was feasible in proton therapy.
• Lifetime coronary event risk decreased by 0.32 % with 3 mm setup uncertainty.
• V95 remained above 98 % at both 4 mm and 3 mm setup uncertainties.
• Mean heart dose decreased from 3.4 Gy(RBE) at 5 mm to 3.0 Gy(RBE) at 3 mm.

## Graphical abstract

**Figure:**
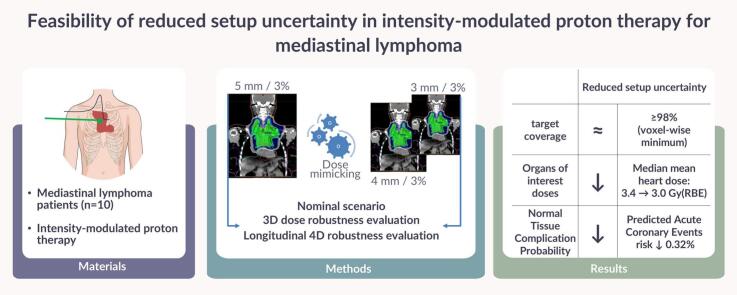

## 1 Introduction

Mediastinal lymphoma patients (HL, primary mediastinal B-cell, or large B-cell lymphoma) with early-stage disease or incomplete response to systemic therapy may receive radiotherapy. However, this relatively young patient population suffers from long term side effects which are related to the dose received by organs of interest (OOI) [1], [2], [3], [4], [5], [6], [7], [8], [9]. To reduce these side effects, technological advances such as intensity-modulated proton therapy (IMPT) are being considered, offering steep dose gradients that spare OOI [10].

In the Netherlands, adult mediastinal lymphoma patients are selected for proton radiotherapy using a model-based selection (MBS) approach applied in routine clinical practice [11], based on differences in the estimated lifetime risk of acute coronary events (ACE) [12] and secondary malignancies between optimal photon and proton plans. Pediatric patients qualify for proton treatment based on the As Low As Reasonably Achievable (ALARA) principle.

IMPT is sensitive to uncertainties, such as range and setup. Robust optimization methods help to create effective treatment plans by covering target volumes while minimizing OOI doses [13]. However, excessively large robust parameters can increase OOI doses without improving outcomes. Studies in head and neck cancer patients have shown that reducing setup uncertainties during plan optimization leads to a greater decrease in normal tissue complication probabilities (NTCPs) compared with reducing range uncertainties [14], [15].

This study aimed to assess whether reducing the setup uncertainty from 5 mm to 4 mm or 3 mm maintains robust treatment plans and improves OOI sparing in mediastinal lymphoma patients treated with intensity-modulated proton therapy.

## 2 Materials and methods

### 2.1 Patients

The patient cohort included in this study consisted of 10 consecutive mediastinal lymphoma patients (both children and adults) treated from 2018 to 2021 with IMPT at our institute. Patients were selected for proton therapy according to the Dutch national indication protocols for proton therapy [11], [16]. Patients younger than 18 are routinely indicated for proton therapy, while adult patients are selected using MBS based on the estimated lifetime excess risk of ACE comparing proton with photon plans. The model estimating the excess risk of ACE is based on the model of Darby et al. and applied to the Dutch population [1], [11]. The input parameters in our MBS are: mean heart dose (MHD), age, gender, and the absence or presence of risk factors for ACE [11]. The ΔNTCP is the excess risk by subtracting the individual risk calculated with MHD with protons from the risk based on the MHD with photons and must be at least 2 % as defined by the health authorities to qualify a patient for the proton treatment.

The treatment volumes for the included patients extended from the neck to the mediastinum, in one case including the left axilla (n = 1), and in most cases encompassed a region surrounding the heart (n = 9), further details described in Table 1. This retrospective study was approved by the Central Ethical Review Committee of the University Medical Center Groningen (160237). All surviving patients were contacted, and informed consent for publication was obtained when possible. For patients who did not return the consent form despite two invitations, their anonymized data were included only after verifying no objection in the national objection registry. This approach conforms to institutional policy and ethical standards for retrospective studies.

**Table 1:** Characteristics of the treatment course preparation specifics for the 10 patients. The point-max motion corresponds to the maximum motion observed for any voxel in the target volume based on the planning 4DCT.

Pt. #	Prescription [Gy(RBE)]	ITV volume [cm^3^]	Mean motion [mm]	Point-max motion [mm]
1	15 × 2.0	339	3.8	20.0
2	15 × 2.0	290	0.4	13.0
3	15 × 2.0	338	4.3	17.0
4	15 × 2.0	317	1.2	6.0
5	15 × 2.0	290	3.7	14.0
6	15 × 2.0	166	2.2	5.0
7	11 × 1.8	259	1.6	6.0
8	11 × 1.8	404	2.0	9.0
9	11 × 1.8	632	1.6	6.5
10	15 × 2.0	251	3.8	10.0

Treatment consisted of 19.8 or 30.0 Gy(RBE)^1^ to the internal target volume (ITV) fractionated respectively in 1.8 or 2.0 Gy(RBE) per fraction with five fractions per week considering a constant relative biological effectiveness (RBE) factor of 1.1. All patients were irradiated in the supine position, with the arms alongside the body and fixation in a 5-point mask (Orfit Industries, Belgium). Prior to each fraction, positioning correction vectors were first determined by matching daily kV-kV images and then daily cone-beam computed tomography (CBCT) images to the plan computed tomography (CT) scan and applied using a 6-D robotic table (BizLink ORION, France) capable of yaw, pitch and roll corrections. IMPT treatments were delivered at our clinical proton treatment facility equipped with the IBA Proteus PLUS system (IBA, Belgium) [17]. Planning and weekly 4DCT scans were acquired using a Somatom AS Open (Siemens Healthineers, Erlangen, Germany) to monitor anatomical changes throughout treatment.

### 2.2 Treatment planning

For each patient, a 10-phase 4DCT was acquired and the ITV was delineated as the envelope of the target contours across all respiratory phases. Then, IMPT plans were 3D robustly optimized on the average 4DCT in the treatment planning system (TPS) RayStation Research v8.99 (Proton PBS Monte Carlo v4.5) (RaySearch Laboratories, Sweden). Two to four beams were used depending on target shape with five-layer repainting, and doses calculated with 1 % statistical uncertainty on 3x3x3 mm^3^ voxels using Monte Carlo. The standard approach was to use three anterior beams (330, 0 and 30 degrees), but in some cases, one of the two oblique beams was not used if the target was predominantly located on one side. In addition, when the target location extended so far caudally to be partially localized posterior of the heart, one or two posterior beams were used to reduce dose to the heart. These beams were then joined to the others by creating a junction region of at least 3 cm (I-S direction). Furthermore, in some patients, the target also extended into the axillary region and a posterior beam was used to treat this area.

Clinical treatment plans were robustly optimized using a 3 % range and 5 mm setup uncertainty: 7 different isocenter shifts (i.e., no shift or a positive or negative shift along one of the three axes) and three (i.e., positive or negative or null) density shifts were considered. The optimization was performed in RayStation by selecting, among the 21 considered scenarios, the scenario resulting in the lowest target coverage. Finally, the multi-scenario voxel-wise minimum approach was used to evaluate the plan robustness as outlined in a recent publication that describes the Dutch consensus for proton plan evaluation [13], [18]. In detail, the robustness evaluation was performed on the average 4DCT used for planning. Fourteen different isocenter shifts (i.e., a positive or negative shift along each of the three axes and along each diagonal) and two density shifts were considered, thus evaluating a total of 28 scenarios [13]. In our clinical practice, we apply a target coverage criterion where D_98 %_ (% of the prescribed dose) must exceed 95 % of the prescription dose and V_95 %_(%) must exceed 98 % in the voxel-wise minimum dose distribution. The required coverage of the delivered dose distribution should be more than 98 %, as the voxel-wise minimum dose distribution is less favorable than any single scenario [15].

### 2.3 Robust planning (3D-nominal) with reduced setup uncertainty and 3D robustness evaluation method (3DREM)

Two additional plans with 4 mm and 3 mm setup uncertainties were generated per patient using an in-house script based on a voxel-based dose mimicking optimization approach [15], maintaining consistent plan quality by prioritizing OOI sparing and the prescribed tumor coverage defined by the physician.

The 3DREM evaluation was performed with the same setup uncertainty parameter used during optimization, reflecting the clinical workflow where robustness evaluation aligns with the assumptions applied in plan optimization. Comparative robustness across different setup uncertainties was then assessed using the 4DREM, which uniformly applies setup, range and machine uncertainties, weekly anatomical variations, breathing motion, and interplay effects to all treatment plans, as described in the following paragraph.

In this study, 3D-nominal refers to the optimized dose distribution on the planning 4D-average CT before treatment. This differs from 3DREM, where 28 scenarios are generated on the 4D-average CT, including ±3 % range uncertainty and fourteen systematic/random setup uncertainties scenarios in x, y, and z directions, depending on the setup uncertainty used during optimization. It also differs from 4DREM, which evaluates the accumulated dose longitudinally during treatment.

3DREM results should be interpreted as a simulation of the planning phase, while the actual robustness comparison of different margins was performed with 4DREM under identical comprehensive uncertainty scenarios.

### 2.4 4D robustness evaluation method (4DREM) applied to our patient plans

To evaluate the robustness of the applied margins, patient-specific longitudinal dose accumulation was performed using our in-house 4DREM [19], which simulates setup and range uncertainties, machine discrepancies, weekly anatomical changes, respiratory motion, and interplay effects. For each patient, eight treatment fractions were simulated, and for each fraction, fourteen independent scenarios were evaluated, including systematic range uncertainties of 0 % or ±3 % and systematic and random setup uncertainties per fraction (total magnitude 2 mm, van Herk formula) [20]. The 2 mm value reflects the residual setup uncertainty after CBCT-based alignment with 6D couch correction.

The dose contribution from each individual spot was calculated on the corresponding weekly 4DCT phase, integrating treatment log files information combined with a 4.5 s breathing cycle. All doses were warped to the end-of-exhale phase of the planning CT using deformable image registration (ANACONDA) and averaged across the eight fractions, each equally weighted over the weekly verification 4DCTs [21]. Voxel-wise mean and minimum dose distributions were then derived to assess both average and worst-case delivered dose, ensuring a realistic robustness evaluation across all uncertainties. These voxel-wise dose distributions were used to evaluate delivered target coverage and OOI doses; 3DREM results were used only to verify planning robustness for each setup uncertainty setting.

### 2.5 Statistical analysis

Differences between the three setup uncertainty configurations (5, 4 and 3 mm) were assessed using the Wilcoxon signed-rank test for paired data. For multiple comparisons, Bonferroni correction was applied. Differences were considered statistically significant if the p-value was below the corrected threshold (α = 0.017). Data are reported as median (range).

## 3 Results

**Fig. 1:**
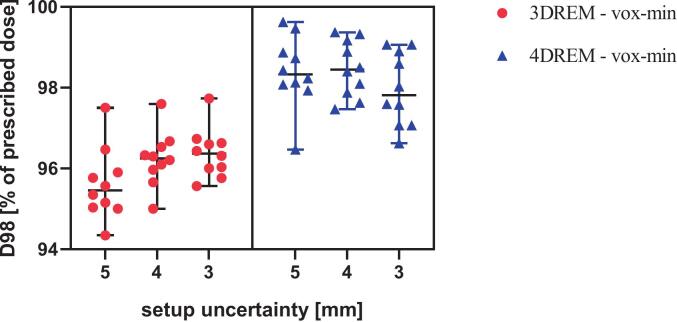
Patient-specific D_98_(%) in the voxel-wise minimum dose distributions for the ITV in 3DREM and the CTV in 4DREM, shown for setup uncertainties of 5, 4, and 3 mm. Horizontal bars represent the median and whiskers indicate the range (minimum–maximum).

**Fig. 2:**
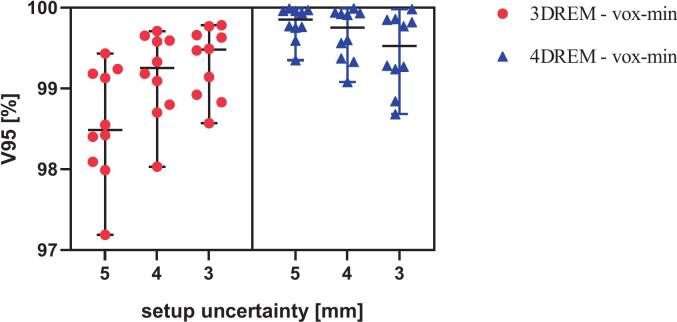
Patient-specific V_95_(%) in the voxel-wise minimum dose distributions for the internal target volume (ITV) in 3DREM and the clinical target volume (CTV) in 4DREM, shown for setup uncertainties of 5, 4, and 3 mm. Horizontal bars represent the median and whiskers indicate the range (minimum–maximum).

The target was adequately covered in all 3D-nominal and 4DREM voxel-wise mean dose distributions, with V_95 %_ > 99.6 % in all plans across the three setup uncertainties. In the voxel-wise minimum dose distributions, target coverage also remained high in both 3DREM and 4DREM, with V_95 %_ > 98 % and D_98 %_ > 95 % of the prescribed dose for all setup uncertainty settings except for one clinically accepted deviation (Table 2). Patient #7 showed a small underdosage, accepted by physician, in the clinical 5 mm treatment plan (D_98 %_ = 94.34 %, V_95 %_ = 97.19 %), evaluated in the voxel-wise minimum dose, which was not observed with 4DREM and also with smaller setup uncertainties. Overall, target coverage remained comparable across all setup uncertainty settings (Table 2). These differences were small and not statistically significant (all p > 0.05). Patient-specific D_98 %_(%) and V_95 %_(%) values for the different setup uncertainties showed only small variations across setup uncertainty settings and are shown in Fig. 1, Fig. 2.

**Table 2:** Target coverage statistics reported as median (range) of the voxel-wise minimum dose distributions for 3DREM and 4DREM (n = 10). Internal target volume (ITV) has been used for 3DREM voxel-wise minimum and clinical target volume (CTV) on reference phase for the 4DREM voxel-wise minimum evaluations (n = 10).

	D_98 %_[% of the prescription dose]			V_95 %_ [%]		
	5 mm	4 mm	3 mm	5 mm	4 mm	3 mm
3DREM	95.4 (94.3–97.5)	96.2 (95.0–97.6)	96.4 (95.6–97.7)	98.6 (97.2–99.4)	99.2 (98.0–99.7)	99.3 (98.6–99.8)
4DREM	98.3 (96.5–99.6)	98.7 (97.5–99.4)	97.8 (96.6–99.1)	99.8 (99.4–100.0)	99.7 (99.1–100.0)	99.3 (98.7–100.0)

**Fig. 3:**
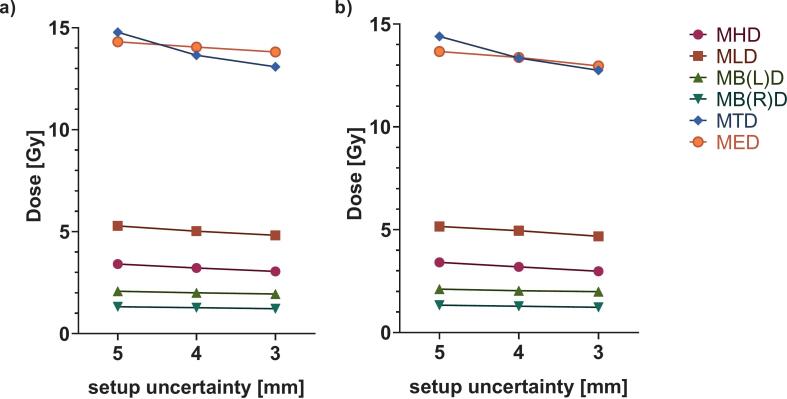
Median organ of interest dose trends as a function of robustness setup: a) 3D-nominal and b) 4DREM. Numerical values are reported as median (range) in Table 3. (abbr. MHD – mean heart dose, MLD – mean lung dose, MB(L)D – mean breast (left) dose, MB(R)D – mean breast (right) dose, MTD – mean thyroid dose, MED – mean esophagus dose).

Dose and volume metrics for OOI showed statistically significant reductions with decreasing setup uncertainty across all pairwise comparisons between 5, 4, and 3 mm (all p < 0.01). As summarized in Table 3 and Fig. 3, the MHD decreased from 3.4 Gy(RBE) (0.1–9.7 Gy(RBE)) at 5 mm to 3.2 Gy(RBE) (0.1–9.4 Gy(RBE)) at 4 mm and 3.0 Gy(RBE) (0.1–9.1 Gy(RBE)) at 3 mm in the 3D-nominal plans, reflecting improved OOI sparing at the planning level, with this trend confirmed by the longitudinal 4DREM results, which decreased from 3.4 Gy(RBE) (0.1–9.9 Gy(RBE)) to 3.2 Gy(RBE) (0.1–9.6 Gy(RBE)) and 3.0 Gy(RBE) (0.1–9.2 Gy(RBE)). This resulted in significant reductions in NTCP for lifetime ACE risk of 0.17 % (0.01–0.31 %) (5 vs 4 mm), 0.32 % (0.01–0.55 %) (5 vs 3 mm) and 0.15 % (0.01–0.28 %) (4 vs 3 mm) (all p < 0.01).

**Table 3:** Median (range) of organ of interest dose and volume statistics for all treatment scenarios under different robustness setup uncertainty criteria (5, 4, and 3 mm) for both 3D-nominal and 4DREM voxel-wise mean dose (n = 10). Abbr. MHD – mean heart dose, MLD – mean lung dose, MB(L)D – mean breast (left) dose, MB(R)D – mean breast (right) dose, MTD – mean thyroid dose, MED – mean esophagus dose.

		Setup uncertainty		
DOSE [Gy(RBE)]		**5 mm**	**4 mm**	**3 mm**
MHD	**3D-nominal**	3.4 (0.1–9.7)	3.2 (0.1–9.4)	3.0 (0.1–9.1)
	**4DREM**	3.4 (0.1–9.9)	3.2 (0.1–9.6)	3.0 (0.1–9.2)
MB(L)D	**3D-nominal**	2.1 (0.2–8.2)	2.0 (0.2–8.0)	1.9 (0.1–7.8)
	**4DREM**	2.1 (0.2–8.1)	2.0 (0.2–7.9)	2.0 (0.1–7.7)
MB(R)D	**3D-nominal**	1.3 (0.1–4.3)	1.3 (0.1–4.1)	1.2 (0.1–4.1)
	**4DREM**	1.3 (0.1–4.2)	1.3 (0.1–4.1)	1.2 (0.1–4.0)
MTD	**3D-nominal**	14.8 (5.6–27.7)	13.6 (5.6–26.6)	13.1 (5.3–26.3)
	**4DREM**	14.4 (6.0–27.4)	13.4 (5.8–26.5)	12.7 (5.6–26.2)
MLD	**3D-nominal**	5.3 (4.1–8.6)	5.0 (4.0–8.2)	4.8 (3.8–7.9)
	**4DREM**	5.2 (4.1–9.0)	5.0 (3.8–8.6)	4.7 (3.6–8.3)
MED	**3D-nominal**	14.3 (4.3–21.2)	14.1 (3.8–20.9)	13.8 (3.6–20.8)
	**4DREM**	13.7 (3.7–21.0)	13.4 (3.4–20.6)	13.0 (3.1–20.4)

VOLUME [cm^3^]				
V_5 %_ LUNGS	**3D-nominal**	31.8 (21.1–47.7)	30.9 (20.4–46.0)	29.7 (19.8–44.6)
	**4DREM**	30.9 (20.8–50.7)	29.8 (20.1–48.5)	28.4 (19.4–47.0)
V_4 %_ L BREAST	**3D-nominal**	16.4 (0.3–68.5)	15.8 (0.2–67.2)	15.4 (0.2–66.4)
	**4DREM**	16.8 (0.3–68.3)	16.2 (0.3–67.0)	15.8 (0.2–66.1)
V_4 %_ R BREAST	**3D-nominal**	12.5 (0.0–39.1)	11.9 (0.0–38.1)	11.5 (0.0–37.6)
	**4DREM**	12.6 (0.0–38.5)	12.1 (0.0–37.6)	11.6 (0.0–37.1)

## 4 Discussion

In this retrospective study, we investigated the impact of reducing setup uncertainty during robust intensity-modulated proton therapy planning for mediastinal lymphoma patients. Using a longitudinal 4D robustness evaluation, this study demonstrated that reducing setup uncertainty to 3 mm was feasible and resulted in consistent OOI sparing without compromising delivered dose robustness. Reducing setup uncertainty from 5 mm to 4 mm and 3 mm led to reduced OOI doses, particularly to the heart, while preserving target coverage. Importantly, longitudinal dose accumulation demonstrated that delivered dose robustness was maintained across all setup uncertainty settings.

This study incorporated anatomical changes, systematic and random setup and range uncertainties to determine the estimated delivered dose through dose mapping and accumulation using longitudinal CT imaging in mediastinal lymphoma patients treated with IMPT. Optimization techniques have been developed to manage uncertainties associated with IMPT, such as range, setup, anatomic changes, dose calculation and NTCP [22]. The robust optimization method has been developed to overcome range and setup uncertainties incorporating them directly into the optimization algorithm [23], [24], [25], [26], [27]. Although widely used clinically [23], [28], [29], excessively large robust optimization parameters may increase OOI doses without improving target coverage [14], [15].

The methods applied to this study offer a more patient outcome driven approach compared to only considering the risk of missing the target, since we employed clinically relevant endpoints (i.e., NTCP) to evaluate the dose distribution retrieved using different setup margins. Although treatment plans generated from scratch with smaller setup uncertainties might yield similar trends, the dose-mimicking approach was deliberately chosen to isolate the effect of setup uncertainty reduction while maintaining identical clinical priorities.

Adequate target coverage was maintained across all reduced setup uncertainties in both the planning-based and longitudinal robustness assessments. To clearly distinguish between planning robustness and delivered dose robustness, it is important to interpret the following results in their appropriate clinical context. It is important to note that the 3DREM analysis in this study reproduced the planning phase of clinical practice, where optimization and robustness evaluation are based on the same setup uncertainty parameter. In this context, the aim was not to demonstrate a systematic improvement in target coverage, but rather to confirm that reducing the setup uncertainty does not compromise coverage, which is already clinically satisfactory. From a clinical perspective, maintaining adequate target coverage while reducing margins is considered a favorable outcome, based on the ALARA principles. The actual robustness comparison across margins was instead performed with the 4DREM, which evaluates all plans under the same comprehensive set of uncertainties and variations.

As shown in Table 2, target coverage, evaluated in terms of voxel-wise minimum V_95 %_(%) and D_98 %_(%), remained high across all setup uncertainty configurations in the 3DREM. Similar observations have been reported in head and neck IMPT, where smaller setup uncertainty settings maintained adequate target coverage while improving normal tissue sparing [15]. The observed differences between setup uncertainty settings reflect the use of different robustness parameters during plan optimization and should therefore be interpreted as a verification of planning robustness rather than as differences in delivered dose robustness. In contrast, the 4DREM, by assessing the accumulated dose longitudinally across the entire treatment course, revealed a slight reduction in target coverage for smaller margins. This decrease arises because the 4DREM comprehensively accounts for multiple sources of variation, including respiratory motion, patient-specific anatomical changes from weekly 4DCTs, setup, range and machine delivery uncertainties, and interplay effects between beam delivery and moving anatomy. Nevertheless, these variations remained within clinical tolerance limits, confirming that reducing setup uncertainty to 3 mm does not compromise treatment robustness.

To our knowledge, the impact of the setup uncertainty parameter on NTCP evaluations has not previously been investigated for mediastinal lymphoma patients, but only for other tumor sites [14], [15]. In our study, a reduction of 0.17 % (0.01–0.31 %) for 4 mm and 0.32 % (0.01–0.55 %) for 3 mm was observed in the 4DREM analysis for the lifetime risk of ACE when compared to 5 mm setup uncertainty. Although small, these reductions were statistically significant and consistent with ALARA, potentially lowering other radiation-induced secondary effects. These findings are consistent with previous studies demonstrating that reducing setup uncertainty during robust optimization can decrease normal tissue doses and associated NTCP values, particularly in head and neck cancer patients [14], [15]. A dedicated study is warranted to verify whether such margin reductions could be applied effectively in photon treatment as well.

Our study has some limitations; first is the limited number of patients involved, but this is closely linked to the generally small number of patients per year presenting with this disease. Second, weekly verification CT scans were used to perform our analysis, but this led to an overestimation of the variability of treatment positioning, as it is generally better during treatment due to the use of various positioning systems. This limitation could be reduced with the introduction of the daily synthetic-CT generated from the daily CBCT acquired on the day of the treatment [30]. Third, the range uncertainties’ impact on plan robustness has not been investigated, but earlier studies showed that the influence of an eventual range uncertainty reduction would be negligible compared to the setup uncertainty reduction, especially for quite superficial targets [15], [31]. Fourth, the model estimating the excess risk of ACE, based on the model described by Darby et al. and applied to the Dutch population, is validated for people older than 40 years. However, since it is currently the only model predicting NTCP for mediastinal lymphoma patients, it is used for all patients, including those under 40, in which case age is considered to be 40, which may lead to an underestimation of NTCP values. Finally, probabilistic setup uncertainties sampling in 4DREM and the small number of fractions (8) may limit reproducibility, but was sufficient to simulate the clinical scenario while balancing computational time and motion-induced effects [19], [32]. In addition, although RayStation research v8.99 was used, this does not affect the comparative nature of the present study, as all plans were generated and evaluated within the same computational framework.

The findings of this study indicate that a 3 mm/3 % setup and range uncertainty configuration provides sufficient robustness for the optimization of mediastinal lymphoma proton treatment plans, when combined with high-precision image-guided positioning using a 5-point immobilization mask, 6D couch corrections, and daily CBCT. This work represents an important step toward the clinical implementation of reduced setup margins in IMPT for mediastinal lymphoma, supporting the feasibility of adopting a 3 mm setup uncertainty without compromising target coverage. Future prospective studies will be essential to further validate these results and confirm their applicability in routine clinical practice.

## CRediT authorship contribution statement

**Pietro Pisciotta:** Writing – review & editing, Writing – original draft, Visualization, Validation, Supervision, Software, Project administration, Methodology, Formal analysis, Conceptualization. **Adriaan Hengeveld:** Writing – review & editing, Writing – original draft, Visualization, Validation, Software, Formal analysis, Data curation. **Erik W Korevaar:** Writing – review & editing, Writing – original draft, Visualization, Validation. **Sabine Visser:** Writing – review & editing, Writing – original draft, Visualization, Validation, Software, Formal analysis, Data curation. **Dirk Wagenaar:** Writing – review & editing, Visualization, Validation, Software. **Petra Klinker:** Writing – review & editing, Visualization, Validation, Software, Formal analysis, Data curation. **John H. Maduro:** Writing – review & editing, Writing – original draft, Visualization, Validation, Data curation. **Johannes A Langendijk:** Writing – review & editing, Validation, Supervision. **Anne GH Niezink:** Writing – review & editing, Writing – original draft, Visualization, Validation, Supervision, Methodology, Conceptualization. **Stefan Both:** Writing – review & editing, Validation, Supervision.

## Declaration of competing interest

The authors declare the following financial interests/personal relationships which may be considered as potential competing interests: The department of Radiation Oncology at the University Medical Center Groningen has researchcollaborations with IBA, RaySearch Laboratories, Siemens, Mirada Medical and VisionRT.

## Footnotes

1 This nomenclature refers to the ICRU 93 definition.
